# Supplementary material for: The genome sequence of the Red Sword-grass moth, Xylena vetusta (Hubner, 1813) (Lepidoptera: Noctuidae)
Source: Wellcome Open Res. 2026 Aug 25;11:545. [Version 1] doi: 10.12688/wellcomeopenres.27382.1 (PMC13586606; doi:10.12688/wellcomeopenres.27382.1)
Supplement: Peer review history

# Reviewer report: Reviewer response for version 1

**DOI:** 10.21956/wellcomeopenres.30137.r166025

**Published:** 2026-09-17

**Authors:** Jonathan Badger

**Affiliations:** 1 Laboratory of Integrative Cancer Immunology (LICI), National Cancer Institute Center for Cancer Research (Ringgold ID: 272101), Bethesda, Maryland, USA

The authors describe their genome assembly from a male *Xylena vetusta*, a moth found in Britain and Ireland. The assembly contains two haplotypes, one with the Z sex chromosome. The sequencing was performed using the PacBio HiFi method with a Hi-C library generated to perform an integrated assembly,  The assembly quality was assessed with a variety of tools such as MerquryFK and BUSCO. The statistics look good and the data is available in the ENA as described.

Are sufficient details of methods and materials provided to allow replication by others?

Yes

Is the rationale for creating the dataset(s) clearly described?

Yes

Are the datasets clearly presented in a useable and accessible format?

Yes

Are the protocols appropriate and is the work technically sound?

Yes

Reviewer Expertise:

I am a computational genomicist, primarily in regard to biomedical projects, although I have also participated in insect genome projects.

I confirm that I have read this submission and believe that I have an appropriate level of expertise to confirm that it is of an acceptable scientific standard.

# Reviewer report: Reviewer response for version 1

**DOI:** 10.21956/wellcomeopenres.30137.r166022

**Published:** 2026-08-31

**Authors:** Mohadeseh Tahami

**Affiliations:** 1 Finnish Museum of Natural History (Ringgold ID: 154275), Helsinki, Uusimaa, Finland

The study provides whole genome assembly of a Xylena vetusta (Hubner, 1813) male moth collected from Scotland, UK, as part of Darwin Tree of Life Project. PacBio HiFi and Hi-C sequencing techniques are applied to build a robust reference genome. The study generates high-quality reads; ~5 million reads for PacBio and 319 million reads for Hi-C with 63x coverage resulting in 2 haplotypes. The genome assembly size is 611.09 Mb with BUSCO completeness 99%.

Are sufficient details of methods and materials provided to allow replication by others?

Yes

Is the rationale for creating the dataset(s) clearly described?

Yes

Are the datasets clearly presented in a useable and accessible format?

Yes

Are the protocols appropriate and is the work technically sound?

Yes

Reviewer Expertise:

taxonomy, NGS, phylogeny, evolutionary genomic

I confirm that I have read this submission and believe that I have an appropriate level of expertise to confirm that it is of an acceptable scientific standard.
